# *Cistus incanus* from Strandja Mountain as a Source of Bioactive Antioxidants

**DOI:** 10.3390/plants7010008

**Published:** 2018-01-26

**Authors:** Vanya Dimcheva, Maria Karsheva

**Affiliations:** Department of Chemical Engineering, University of Chemical Technology and Metallurgy, 8 Kl. Ohridski bul., 1756 Sofia, Bulgaria; mik@uctm.edu

**Keywords:** *Cistus incanus*, Strandja, antioxidants, polyphenols, flavonoids, seasonality, buds, hard-coated seeds

## Abstract

The purpose of the present study is to survey the extraction conditions and explore the antioxidant potential of the wild herb *Cistus incanus*, which is non-traditional in Bulgarian ethnomedicine and widespread in the Strandja Mountain. The influence of the extraction time (0–500 min) and solvent composition (0–50% ethanol in water) on the polyphenols, flavonoid yields and on the antioxidant capacity of the extracts of leaves, stalks (wood parts) and bud mixture were studied. The antioxidant capacity (AOC) was evaluated by use of scavenging assays of 2,2-diphenyl-1-picrylhydrazyl (DPPH) radicals. Total polyphenol and flavonoid contents were quantified using UV–vis (ultraviolet-visible) spectrophotometry. The optimal yield of the desired components was obtained with 30% ethanol in water solvent at the 390th min of extraction time. In addition, the influence of seasonality (winter and summer *Cistus incanus*), and of the different aerial parts—hard-coated seeds, buds, and a mixture of leaves and stalks of the wild plant—on the presence of polyphenols, flavonoids, and AOC were investigated. The present work revealed that the high values of polyphenols, flavonoids and the high AOC occurred not only in the summer leaves, but were also found in the winter leaves, hard-coated seeds, buds, and stalks. Based on the obtained results, the *Cistus incanus* from Strandja Mountain could be an excellent new source of natural antioxidants in food and for the pharmaceutical industries.

## 1. Introduction

Medicinal plants, especially with antioxidant activity, are the main source of drugs for the treatment of complications induced by oxidative stress. Today, about half of the available drugs are estimated to come from plants [1]. The synthesized drugs may appear to have different adverse effects [2]. Therefore, it is important to look for new sources of phytomedicines in nature.

*Cistus incanus* L. has a habitat of temperate ericoid communities or European dry heaths, and in Bulgaria covers almost the whole of the most intriguing region, the Strandja Mountain [3]. The plant is not included in the “law of medicinal plants”, nor is it protected or declared as a medicinal plant.

In the Quaternary, among the parts of Europe least affected by glaciation, only the Strandja seaside remains almost untouched and has climatic conditions similar to the tertiary “eternal spring” [4]. Many plants grown here with therapeutic actions are not commonly used and popular in Bulgarian folk medicine, such as the sub-endemic *Cistus incanus* L. or “Pamukliyka” (local name). It is most well-known only as food for goats and sheep in this area, but the history of the “Holly rose” and its ethnomedicinal usage in the Mediterranean began in ancient times [5]. The wild herb has provided antibacterial, antimicrobial, anti-inflammatory and strong gastroprotective beneficial effects [6]. Many research studies have demonstrated that the main components of the leaves of the different *Cistus* species are polyphenolic compounds from flavanols, a flavan-3-ols family such as (+)-catechins, gallic acid, rutin, flavonoid aglycones based on quercetin, kaempferol, and myricetin [7,8]. It is well established that the phenolics content in plants is mainly responsible for their antioxidant activities and scavenging power. 

This work contributes to establishing the beneficial properties of the Bulgarian *Cistus incanus*. The evergreen herb has the tendency to polymorphism or alteration of the phytochemical composition under different environmental factors, conditions, and seasons. An appropriate extraction of phenolic compounds depends on multiple factors, such as their chemical nature, raw material, storage time and conditions. Not least it depends on the extraction and quantification methods, choice of standards, and presence of interference [9,10]. Thus, it is necessary to adjust the sample preparation procedures to achieve the optimal estimation of the phenolic compounds. The results from the evaluation of the operational extraction conditions of the *Cistus incanus* will provide a better understanding of the antioxidant potential of the wild herb and will allow its use as a high-added-value dietary antioxidant additive.

In this investigation, we elected to follow the steps of the extraction optimization of *Cistus incanus* by total polyphenols, flavonoids, and antioxidant capacity. Initially, the effect of the solvent (ethanol in water mixtures) concentration was evaluated for a previously chosen extraction time. Once the optimal solvent concentration was found, the extraction time at the constant, previously-chosen extraction parameters was evaluated, namely temperature, particle size, and solid-to-solvent ratio. This was also followed by studying the kinetics by the total dry residue of the extracts, the kinetics by total dry mass, and the kinetics by the final volume of the extracts received after hand pressing the exhausted plant material. The kinetics were carried out to establish the equilibrium of the extraction process for a better understanding of the extraction process of the herb studied. In addition, the influence of seasonality and the evaluation of the different aerial parts of *Cistus incanus* for the presence of polyphenols and flavonoids was also investigated.

## 2. Materials and Methods

The total polyphenol content (TPC) was determined through the Folin–Ciocalteau method at a wavelength of 765 nm. The total flavonoid content (TFC) was measured by aluminum chloride colorimetric assay at a wavelength of 510 nm. The AOC was studied by DPPH assay at a wavelength of 517 nm. The total dry residue (TDR) was found gravimetrically after the evaporation of 10 mL of the extract and through drying of the exhausted drug to a constant weight in the oven at 105 °C. 

### 2.1. Chemicals

Ethanol 96% was supplied by Valerus (Sofia, Bulgaria). Methanol (gradient grade for liquid chromatography), sodium carbonate (>99%), gallic acid anhydride (>99%), sodium nitrite, and aluminum chloride hexahydrate was supplied by Merck (Sofia, Bulgaria). Folin–Ciocalteau reagent, 2,2-diphenyl-1-picrylhydrazyl (DPPH), rutin hydrate, quercetin hydrate (≥95%), tannic acid (≥91%), pyrogallic acid (≥98%), (+)-catechin hydrate (≥96%), sodium hydroxide, and Trolox (6-hydroxy-2,5,7,8-tetramethylchroman-2-carboxylic acid) (97%) were supplied by Sigma Aldrich (Sofia, Bulgaria). Ammonia iron alum was supplied by Scharlau (Sofia, Bulgaria). Deionized water was from a “Elix70C Gulfstream” water deionizer supplied by Merck (Germany).

### 2.2. Plant Material 

This study used wild *Cistus incanus* L. leaves, stalks (wood parts) and buds collected at the end of May (2015) during the beginning of flowering, hard-coated seeds of the plant collected in September (2015), and leaves and stalks collected at the end of March (2016). The drugs were gathered from the area of Parnara, around the village Varvara (Tsarevo municipality), according to the rules of conservation of the biodiversity of the National Park Strandja, Bulgaria. The temperatures measured on the days of collection of *Cistus incanus* were 25 °C in May 26 °C in September, and 7 °C in March. To ensure a representative sampling, 2 kg of the wild plant were collected. The *Cistus incanus* L. was identified by experienced biologists from the National Park Strandja.

### 2.3. Extraction Procedure

The experiments used the following mixtures of *Cistus incanus*: leaves, stalks, buds (80:10:10, *w*/*w*); *Cistus incanus* stalks and leaves (50:50, *w*/*w*); hard-coated seeds; leaves and stalks (90:10, *w*/*w*) gathered in the summer and winter harvest seasons. All samples were dried at room temperature and kept in a dry place for a year before being ground in the grinder and sieved. All samples were used with a LOD (loss on drying) of not more than 10%. For the experiments, a fraction of 0.5–2.0 mm particle size was used. The initial solid-to-solvent ratio was fixed at 1:20 (2 g *Cistus incanus* in 40 mL solvent). The temperature used for the extraction was room temperature and was kept constant as far as possible. Extractions were done through magnetic stirring at 1411 RCF (relative centrifugal force) with a magnetic stirrer (MS-H-Pro+, Dragon Laboratory Instruments, Beijing, China). The influence of the solvent composition water or water–ethanol solution (10, 20, 30, 40, 50, *v*/*v*) were studied at the 80th min extraction time. The extraction kinetics of the *Cistus incanus* samples were followed for 8.3 h (5, 10, 30, 50, 80, 120, 180, 390, 500 min) with the chosen constant extraction condition. Each exhausted plant material was carefully pressed, and the extract was filtered through cotton and filter paper, measured and analyzed immediately after the appropriate dilution. 

### 2.4. Total Polyphenol Assay by the Folin–Ciocalteau Method 

A volume of 0.1 mL of Folin–Ciocalteau reagent was added to a tube, containing 0.02 mL of the extract (previously diluted to 150 mL/L) and 1.58 mL of deionized water. A minute later, 0.3 mL of a 20% Na_2_CO_3_ solution was added to the tube. The samples were kept in a dark place for 2 h and then the absorbance was measured at 765 nm against the reagent blank with a UV-vis spectrophotometer (T60UV/VIS, Oasis Scientific Ltd, South Carolina, USA) using a 10 mm path length cuvette [11]. The results were calculated as gallic acid equivalents (y = 0.9119x, R^2^ = 0.9892), pyrogallic acid equivalents (y = 1.2114x, R^2^ = 0.9907) and tannic acid equivalents (y = 0.4601x, R^2^ = 0.912). The standard calibration curves were obtained with the following standard solution concentration diapasons: a gallic acid solution (0.1–1.0 mg/mL), a pyrogallic acid solution (0.1–0.75 mg/mL), and a tannic acid solution (0.5–2.0 mg/mL). The total phenolic contents of the *Cistus incanus* extracts was expressed as mg of gallic acid, pyrogallic acid, and tannic acid equivalent per gram dry weight sample (mg GAE, PGAE, TAE/g dw) and calculated by the following formula:

TPC=C × V × F/M

where TPC is the total polyphenol content, mg GAE/g dw, mg PGAE/g dw, and TAE/g dw; C is the concentration of the used standard, mg/mL; V is the volume of the used solvent, mL; F is the dilution coefficient of the sample; and M is the mass of the sample, g.

### 2.5. Flavonoid Assay

The total flavonoid content (TFC) of the plant extracts was expressed as quercetin, rutin, and (+)-catechin equivalents and measured by the aluminum chloride colorimetric assay [12]. An aliquot of 1 mL extract (previously diluted to 150 mL/L) was mixed with 4 mL of deionized water and 0.30 mL of a NaNO_2_ solution (10%, *w*/*v*). At the 6th min, 0.30 mL of an AlCl_3_ solution (10%, *w*/*v*) was added, followed by 2.0 mL of an NaOH solution (1 M). Immediately after thorough mixing, the absorbance was measured at 510 nm versus the blank sample. The calibration curves of the used standards were obtained with a quercetin (100–1000 mg/L; y = 0.000552x; R^2^ = 0.9977), a rutin (20–100 mg/L; y = 0.00115x; R^2^ = 0.9958) and a (+)-catechin (10–200 mg/L; y = 0.00345x; R^2^ = 0.9968). The results are expressed as quercetin, rutin and (+)-catechin equivalents per gram dry weight (mg QE, RE, CE/g dw) and calculated by the following formula:

TFC = C × Ve × F/M

where TFC is the total flavonoid content, mg QE/g dw, mg RE/g dw, mg CE/g dw; C is the concentration of the used standard, mg/L; Ve is the volume of the used solvent, L; F is the dilution coefficient of the sample; and M is the mass of the sample, g.

### 2.6. Antioxidant Activity by the DPPH Method

This is the most commonly used method for the quantification of antioxidant activity. The method was described by Brand–Williams et al. [13] and was later changed by Sánchez–Moreno et al. [14]. DPPH solutions show high absorption at 517 nm due to the deep violet color. The absorbance gradually disappears because of discoloration, which is stoichiometric to the degree of reduction of free radicals. The remaining DPPH measured after a certain time inversely corresponded to the free radical scavenging ability of the antioxidants.

One thousand microliters of various concentrations of the extracts in ethanol were added to 4 mL of a 0.004% methanol solution of DPPH. After an hour’s incubation period at room temperature, the absorbance was measured against a methanol as a blank at 517 nm. Antioxidant activity defined as the extract concentration necessary to neutralize 50% of free DPPH radicals; IC_50_ was calculated by plotting the correlation between the concentration of the extract (μg/mL) and IC (%)—C/IC. The graph was constructed by preparing a series of extracts with various concentrations (0.05–0.25 μg/mL). The free radical scavenging ability of the tested samples was calculated using the formula (Yen and Duh) [15]:

IC=(Ao−Aa)/Ao×100

where IC is the inhibition capacity, %; Ao is the value of absorbance blank; and Aa is the value of absorbance of the sample.

After recalculation, the IC (%) were expressed as the IC_50_ values in μg/mL.

The results derived were also recalculated using Trolox (6-hydroxy-2,5,7,8-tetramethylchroman-2-carboxylic acid), which is an antioxidant vitamin E derivative. It is regularly used as an antioxidant standard. The TEAC (Trolox equivalent antioxidant capacity) assay is often used to measure the antioxidant capacity of foods, beverages and nutritional supplements [16].

The calibration curve of the Trolox was used at a linearity range of 2.5–175 μmol/L. The obtained equation of rights was y = 1.332x + 0.5634, where y is the concertation of the Trolox solution (plotted in the ordinate), and x is the absorbance (plotted in the abscise). The TEAC was calculated using the formula:

TEAC = IC sample−1.332/0.5634

where TEAC is the Trolox equivalent antioxidant capacity, μmol TEAC/L; IC is the inhibition capacity of the sample, %; 1.332 is the slope from the Trolox calibration curve; and 0.5634 is the cut obtained from the Trolox calibration curve.

The data obtained were expressed in μmol TEAC per gram dry weight (μmol TEAC/g dw) of the extracts, using the mass of the samples and the dilution factor.

### 2.7. Total Dry Residue of Extracts

The total dry residue of the extracts was determined in accordance with the Ph. Eur. (European Pharmacopeia) method with some modifications [17]. The exhausted drug and 10 mL of the extract to be examined were introduced rapidly into flat-bottomed dishes. The samples were dried at 105 °C in an oven Determ (Robotica, Velingrad, Bulgaria) to a constant mass. Thereafter, the samples were cooled in a desiccator under anhydrous silica gel and were weighted. The results were calculated as gram per liter.

## 3. Results and Discussion

Detailed literature research into the phenolic compounds present in *Cistus incanus* was carried out. There are no data available concerning kinetic studies of the selected drug by total polyphenol and flavonoid content, antioxidant power, and total dry residue, except the extraction kinetics presented by Dimcheva and Karsheva [18] for Bulgarian *Cistus incanus* leaves with 50% ethanol in water solution. In addition, the influence of seasonality on the presence of the examined bioactive components and thus the AOC of the wild herb has never been studied. There are no data in the literature concerning the polyphenol content and the AOC of the aerial parts (stalks, buds, hard-coated seeds) of *Cistus incanus*.

### 3.1. Effect of the Solvent Used

The conventional method of polyphenol recovery from the plant is based on solid–liquid solvent extraction. It is generally known that the yield of extracted polyphenols depends on the chemical composition and physical characteristics of the samples as well as on the type of solvents used, their different polarity, extraction manner, contact time and temperature. The results can vary even by one order of magnitude when one or another procedure is used for the same sample. Thus, it is necessary to adjust the extraction method for each new crude drug.

Solvents such as methanol, ethanol, acetone, ethyl acetate and combinations of them are most commonly used to extract phenolics from plants, often in different ratios with water. Choosing the right solvent is essential for the industry, it must be safe, cheap and non-toxic. Ethanol is a good solvent for the extraction of polyphenols and preferable for the extraction of *Cistus incanus* according to a patent publication for *Cistus incanus* extracts [19]. Therefore, the ethanol was chosen as the solvent in the present investigation.

**Figure 1:**
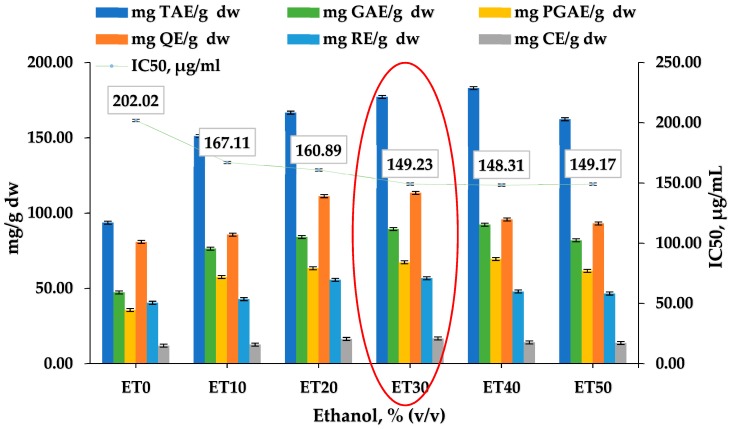
Effect of the solvent concentration on the extraction of *Cistus incanus* leaves, stalks, and buds at the 80th min on Total polyphenol content (TPC), Total flavanoid content (TFC), and Inhibition capacity at 50% (IC_50_).

To be evaluated, the appropriate solvent composition was used a deionized water or an ethanol in water solution (10–50%, *v*/*v*) to establish the optimal yield of total polyphenols, flavonoids, and antioxidant capacity. The extractions were done by a magnetic stirring for 80 min. The results for TPC, TFC, and IC_50_ are shown in Figure 1.

The pure deionized water (ET0) showed the worst values for the desired components at the expense of the medium polar mixtures, such as ET30 and ET40. It can be seen from Figure 1 that the IC_50_ rests constant with the decrease of the polarity of the extracting solvent. Of these, the high values of the extracted antioxidant substances and the highest value of the IC_50_, the ET30, was chosen as the optimal solvent concentration and used in the further examinations.

The present investigation evaluated an extraction parameter—the extraction time (0–500 min)—on total polyphenols, total flavonoids and the scavenging activity of the *Cistus incanus* leaves, stalks, and buds. The temperature and the solid-to-solvent ratio were kept constant during the whole extraction kinetics procedure, which was carried out through magnetic stirring extraction and with ET30 as a solvent. In conventional methods, sampling is manual at chosen time intervals, which are not precise, as there is always a time gap between sampling and analysis that may lead to errors during the kinetic measurements. Nevertheless, in the present study, we tried to make the interval between the various extractions relatively small. On the other hand, the measured raw material was kept as far as possible to the same ratio of leaves, stalks, and buds. The evaluation of the extraction time was investigated on TPC, TFC, IC_50_, and the total dry residue, all shown below.

### 3.2. Total Polyphenols

Phenolic compounds act as essential metabolites for plant growth and reproduction, and as protecting agents against pathogens. These compounds involve a large group of about 8000 compounds with different structures and chemical properties [20]. In general, these substances contain one or more aromatic rings with one or more hydroxyl groups and can be classified into three main categories: simple phenols, which include phenolic acids; polyphenols, constituted by flavonoids and tannins; and a miscellaneous group that comprises compounds such as coumarins, stilbenes, and lignans. 

The total polyphenol content for the 30% ethanol extracts was estimated by the Folin–Ciocalteau method using gallic, pyrogallic and tannic acids as standards. 

Gallic acid is commonly used in the pharmaceutical industry to determine the total phenol content by Folin–Ciocalteau assay [21]. Phenolic acid is mostly used to express the content of phenolic compounds in most foods [22]. On the other hand, pyrogallic acid is used as a standard for the determination of the total polyphenols according to the Eur. Ph. [23]. Similarly, to previous compounds, tannic acid was proved to possess antioxidant [24], antimutagenic [25] and anticarcinogenic properties [26]. 

According to the obtained results, a statistically significant effect of the time of each extract is presented in Table 1, where the content of total polyphenol compounds is shown, expressed by the phenolic acids represented above.

The total amount of polyphenols, expressed as the gallic acid in the extracts, varies between 36.26 and 115.32 mg GAE/g dw as a function of time. The quantity of the polyphenols expressed as tannic acid equivalents ranged from 71.88 to 228.56 mg TAE/g dw and from 27.30 to 86.81 for the PGAE/g dw. Lower phenolic contents were detected at the 5th min and the highest at the 390th min, as shown in Table 1. However, it can be concluded that the equilibrium was achieved at the 180th min because the obtained values for the desired polyphenols were only 4.7% less than those obtained after a 3.5 h extraction and 0.41% less than those obtained after 5.3 h of stirring.

The used Folin–Chiocalteau assay is specific not only to polyphenols but to any other substance that could be oxidized by the Folin reagent; many non-phenolic compounds like ascorbic acid and saccharides can reduce the amount of reagent [14].

### 3.3. Total Flavonoids

Flavonoids are low molecular weight polyphenolic secondary metabolic compounds, universally distributed in the green plant kingdom [27]. Flavonoids represent a broad family of more than 4000 secondary plant metabolites such as 4-isoflavonoids (flavones and flavonols), isoflavones, anthocyanins, and flavan-3-ols derivatives (tannins and catechins) [28]. For centuries, preparations that contain flavonoids have been applied as the primary physiologically active components used for treating human diseases [29].

Quercetin, rutin and catechin are important bioflavonoids present in more than twenty plant materials and are known for their anti-inflammatory, antihypertensive, and vasodilator effects, as well as their antiobesity, antihypercholesterolemic and antiatherosclerotic activity [30,31,32,33].

The total flavonoid content of the ethanolic extracts was measured using an aluminum chloride colorimetric assay using quercetin, rutin and (+)-catechin as standards. Aluminum chloride forms acid stable complexes with the C-4 keto groups and either the C-3 or C-5 hydroxide group of the flavones and flavonols. In addition, it also forms liable complexes with ortho dihydroxide groups in the A/B rings of flavonoids. 

The extraction kinetics data for different species are shown in Table 2.

The total flavonoids, expressed as the quercetin equivalent of the extracts, show higher values varying between 40.80 and 119.20 mg QE/g dw from the 5th to the 500th min extraction time. The lower quantities of the flavonoids were calculated as the (+)-catechin equivalent ranging from 6.0 to 17.53 mg CE/g dw and the middle ones ranged from 20.40 to 59.60 mg RE/g dw for the flavonoids calculated as the rutin equivalent. In Table 2, the presented kinetics show the same tendency as the total polyphenol equilibrium achieved at the 180th min, but the difference here is their decrease after the 390th min. Probably, this decrease is due to their unstable nature or to error due to the time between experiments and other random factors.

No previous study of the kinetics of the total content of the polyphenols and flavonoids in *Cistus incanus* exists, including Bulgarian *Cistus incanus,* as was already mentioned. Hence, the data obtained can only be compared with that found for the *Cistus incanus* species grown in different regions and extracted using different extraction procedures and different conditions to those used in the present study.

For example, for the aqueous extracts of *Cistus ladanifer* and *Cistus populifolius* from Spain, the TPC values at levels of 229.3 mg GAE/g dw and 318.9 mg GAE/g dw, respectively, were found. The TFC values in these plants were found to be 30.4 mg QE/g dw and 59.5 mg QE/g dw, respectively [34].

Similarly, the results for TPC obtained for aqueous extracts of Turkish *Cistus laurifolius* were 289.9 mg GAE/g extract [35].

Lower levels of TPC and TFC were reported for methanol and ethanol extracts of Moroccan *Cistus ladanifer*: 18.43 mg GAE/g extract and 64.33 mg RE/g extract; and 11.87 mg GAE/g extract and 61.40 mg RE/g extract, respectively [36].

In another study of the extracts obtained from *Cistus incanus* grown in Turkey and Cyprus, the following values for the valuable components were obtained: 258.42 mg GAE/g dw and 202.95 mg GAE/g dw for the aqueous extracts, and 105.02 and 114.18 mg GAE/g dw for the hydromethanolic extracts for the total polyphenols content. The total flavonoids for the same extracts were 4.27 and 3.97 mg QE/g dw and 2.39 and 2.27 mg QE/g dw, respectively [37].

From the research it can be concluded that the Bulgarian *Cistus incanus* contain the greatest total flavonoid content (138.44 mg QE/g dw and 69.22 mg RE/g dw) in comparison not only with *Cistus ladanifer* from Morocco and *Cistus populifolius* from Spain and Turkey, but in comparison with the Turkish and Cyprian *Cistus incanus* leaf extracts. The results found in the literature for the total polyphenols of the *Cistus* species are higher than those obtained in this study for the *Cistus incanus* leaf, stalk, and bud hydroethanolic extracts. The quantities of extracted polyphenolic compounds in the plants depend on the differences in extractive parameters and the solvent used. The various biological and environmental factors in which the plant grew also contribute to the plant antioxidant power [38]. 

### 3.4. Antioxidant Capacity

It is well established that the flavonoids and phenolic acids have antioxidant activities due to the presence of structural hydroxyl groups significantly contributing to protection against the oxidative damage due to endogenous free radicals [39,40]. Many of them are reported to have high levels of antioxidant activity [41]. Due to their redox properties, these compounds contribute to the overall antioxidant activity of plants. Usually, the antioxidant activity neutralizes lipid free radicals and prevents the decomposition of hydroperoxides into free radicals [42].

**Figure 2:**
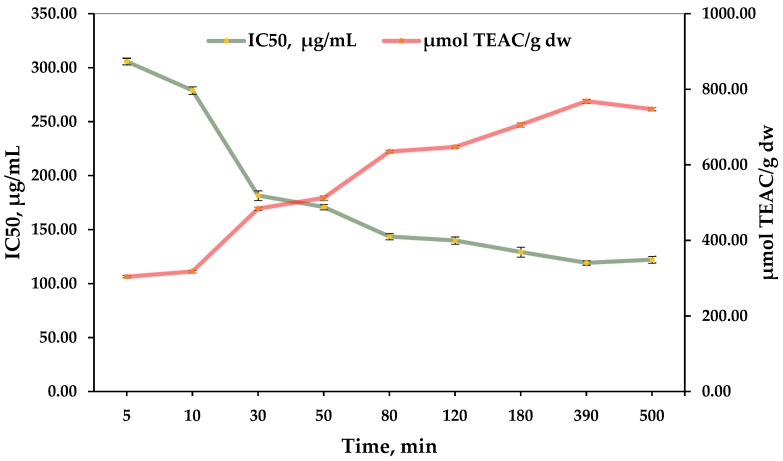
Kinetic curves by IC_50_ in μg/mL and μmol TEAC/g dw of *Cistus incanus* leaf, stalk, and bud extracts.

The IC_50_ and TEAC are presented in Figure 2. Expressed as the concentration of the extract, they vary from 305.71 to 122.16 μg/mL; expressed as a Trolox equivalent, they vary from 303.88–747.13 μmol TEAC/g dw. The best values for the IC_50_ and TEAC of the *Cistus incanus* leaves, stalks, and buds were obtained at the 390th min and were 768.44 μmol TEAC/g dw or 119.25 μg/mL of the extract can reduce 50% of the free radicals.

The literature study found data on 15 different samples of *Cistus incanus* from different countries. The results showed that the values of DPPH for hydromethanolic and aqueous extracts were varied in the range of 20.06–96.69 μmol TEAC/g dw and 1.52–96.85 μmol TEAC/g dw, respectively.

These results are much lower than those obtained in the present study. That means that the Bulgarian *Cistus incanus* is a rich source of antioxidants and the environmental factors of Strandja Mountain are obviously suitable for their formation.

### 3.5. Total Dry Residue

**Figure 3:**
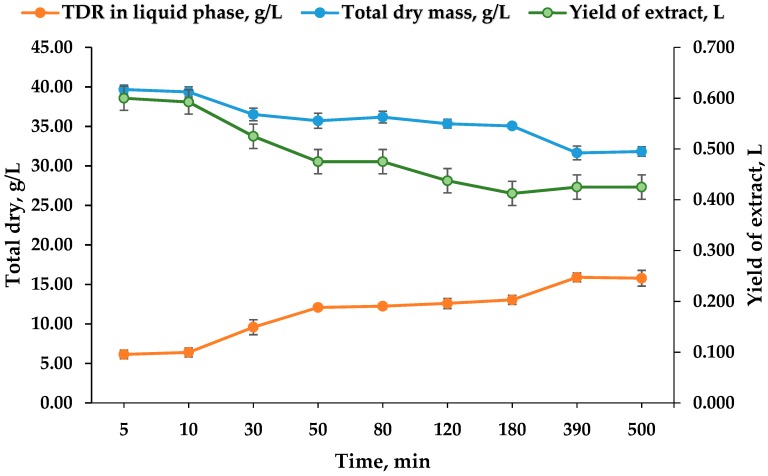
Kinetic curves by the total dry residue (TDR) in the liquid phase, the total dry mass in g/L, and the yield of the extract received after extraction (L) of the *Cistus incanus* leaves, stalks, and buds.

In the evaluation of plant extracts, it is good to know the kinetics of the process also by total dry residue when equilibrium is achieved, not least for a better understanding of the plant material extraction. Using the gravimetric method described above, the kinetics of the total dry residue (TDR) of the *Cistus incanus* leaves, stalks, and buds picked during the summer harvest season in the liquid phase and their total dry mass were studied. Extracts and exhausted plant materials from the extraction kinetic with 30% ethanol and a 0.05 g/mL solid-to-solvent ratio were used. The quantity of the extracts after the hand pressing of the plant material were measured and plotted in the graph. The results for TDR and total dry mass were expressed in grams of dry weight per liter v/s extraction time. The measured volumes of the received extracts were expressed in liters. The yield of the extracts was studied because it is an essential parameter for the industrial production of extracts. The kinetics curves obtained are shown in Figure 3.

In the kinetics presented, the water contents (9.70%) was not recalculated and thus the presence of volatile substances is quite probable. As shown, the kinetic curves have three parts with different characters. The increase of TDR in the liquid phase (extract) corresponds to the decrease of the total dry mass. The initial steep part of the graphic corresponds to the dissolution of the readily available substances on the surface of the sample particles. The second curved part could be explained by the simultaneous dissolution of the rest from the surface and from inside the sample particle (the mixed zone control). Based on the yield of extract kinetic, the plateau or the extraction equilibrium was achieved after the 180th min, where the quantity of the dry extract was 5.38 g in 413 mL extract, but increased to 6.7 g in 425 mL extract at the 390th min. Likewise, there was an increase after the 180th min illustrated by the TDR kinetic responsible for the liquid phase, and the highest results for this kinetic can be seen at the 390th min. However, based on the total dry mass, the plateau was reached approximately at the 80th min, where the quantity of total dry residue of the extract was 1.4135 g and slowly decreased with 1.0% up to the 500th min. These results may be due to the uneven plant material used or measurement errors. Based on the kinetics by total polyphenols, flavonoids, and AOC, it can be concluded that the 390th min, or 6.5 h, is the optimal extraction time also in relation to the yield of the extract and the TDR in the liquid phase. The long extraction time probably shows that magnetic stirring is not the best way to extract the examined mixtures of drugs or that there are bioactive substances in the hard buds and stalks which need more time for their discharging. In both cases, further extraction optimization is required, maybe by increasing the extraction temperature or changing the applied extraction manner. 

### 3.6. Evaluation of Cistus incanus Aerial Parts

In this study different aerial parts were used, as follows: hard-coated seeds and young buds, as well as a mixture of stalks and leaves (50:50%, *w*/*w*). They were extracted for 80 min with 30% ethanol in water solution.

**Figure 4:**
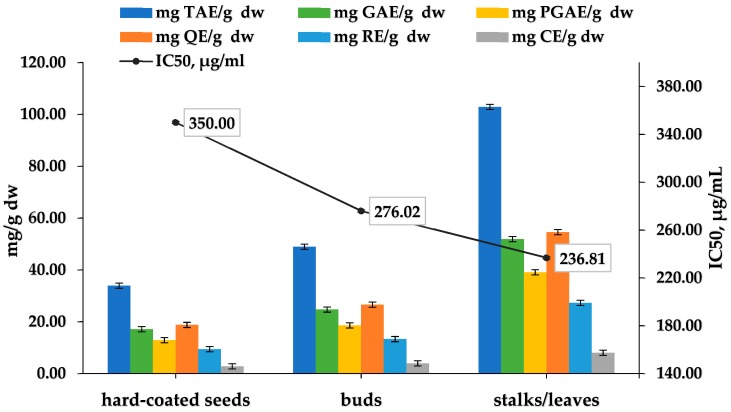
Evaluation of the TPC, TFC, and IC_50_ of the *Cistus incanus* hard-coated seeds, buds and a mixture of stalks and leaves.

The total polyphenols and flavonoids in the buds and in the hard-coated seeds provided good results. The buds should have contained much more of the desired components than the seeds, because they were picked during the plant’s flowering, when it is in its polyphenol power. It is known that the woody parts of the aromatic herbs also contain flavonoids and polyphenols, which play an important role in protecting the plant. [43] As shown in Figure 4, the mixture of leaves and stalks in a ratio 50:50 provided the best results, which is normal because the main quantities of polyphenols are concentrated in the leaves. The obtained results show that the hard-coated seeds, buds, and stalks can also be used as a raw material for the production of antioxidants in the nutraceutical industry or for making a tea (infusion) at home.

### 3.7. Evaluation of Cistus incanus Winter and Summer Leaves

**Figure 5:**
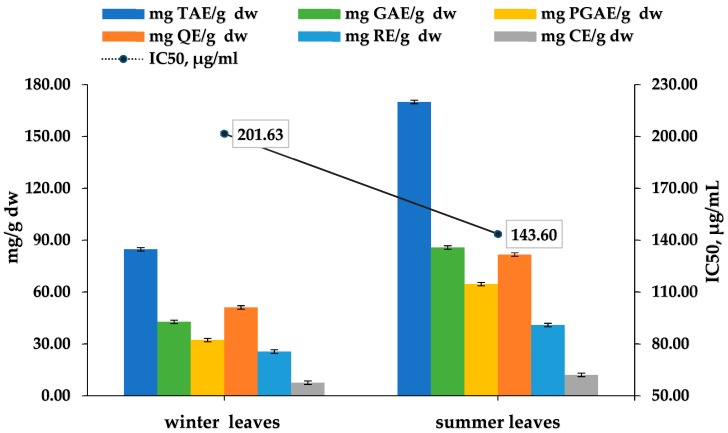
Evaluation of the TPC, TFC, and IC_50_ of the *Cistus incanus* summer and winter leaves.

This study compared mixtures in a mass percent concentration of 90:10 of the *Cistus incanus* leaves and stalks collected in the summer and winter harvest seasons by the yield of antioxidants. The samples were extracted for 80 min with 30% ethanol in a water solution. It is known that the wild plant is an evergreen shrub which blooms from May to September; it is assumed that this is when the flavonoids and polyphenols reach their highest value. The data from other authors about the polyphenolic content and AOC of *Cistus incanus* gathered through the winter is missing. This can be confirmed by the results obtained and summarized in Figure 5. 

Summer *Cistus incanus* leaf and stalk extract gave better IC_50_-143.60 μg/mL or 579.70 μmol TEAC/g dw. The extract of the winter sample provided as good results for the IC_50_-201.63 μg/mL or 377.93 μmol TEAC/g dw. This means that the *Cistus incanus* from Strandja should be collected and used even during the winter season. 

## 4. Conclusions

It can be concluded that the sub-endemic plant, *Cistus incanus*, growing in the entire Strandja mountain, contains high value bioactive components, not only detected in summer or winter leaves, but also in its stalks (wooden parts), buds and hard-coated seeds. The results showed that 30% ethanol in aqueous extracts gave the highest content of total polyphenols and flavonoids, albeit with prolonged extraction. Additionally, the antioxidant activities were well-correlated with the contents of the extracted bioactives. 

This study is an initial step of the extraction evaluation of *Cistus incanus*. Further optimization is possible and necessary for total process evaluation, for example, decreasing the size of particles, changing the extraction manner, or increasing the extraction temperature. The optimization of extraction is required to decrease the obtained long extraction times with respect to increasing costs.

Our results provide a better understanding of the high-value antioxidant potential of the Bulgarian *Cistus incanus* to be applied in the food, cosmetic, and drug fields.

## Figures and Tables

**Table 1:** Total polyphenol kinetic expressed as tannic acid, gallic acid, and pyrogallic acid equivalents in mg per g dry weight of the *Cistus incanus* leaf, stalk and bud extracts.

Extraction Time, min	mg PGAE/g dw ± SD *	mg GAE/g dw ± SD *	mg TAE/g dw ± SD *
5	27.30 ± 1.34	36.26 ± 1.76	71.88 ± 3.48
10	35.67 ± 1.20	47.38 ± 1.62	93.91 ± 3.21
30	52.07 ± 2.77	69.17 ± 3.65	137.09 ± 7.22
50	68.47 ± 2.45	90.95 ± 3.23	180.27 ± 6.40
80	67.26 ± 1.20	89.35 ± 1.62	177.08 ± 3.20
120	75.07 ± 2.05	99.73 ± 2.71	197.66 ± 5.36
180	82.89 ± 0.95	110.11 ± 1.25	218.24 ± 2.47
390	86.81 ± 2.35	115.32 ± 3.10	228.56 ± 6.14
500	82.55 ± 1.95	109.66 ± 2.57	217.34 ± 5.08

* Extractions were performed in duplicate and the results are expressed as the mean ± standard deviation (SD).

**Table 2:** Total flavonoid content expressed as quercetin, rutin and (+)-catechin equivalents in mg per g dry weight of the *Cistus incanus* leaf, stalk, and bud extracts.

Extraction Time, min	mg QE/g dw ± SD *	mg CE/g dw ± SD *	mg RE/g dw ± SD *
5	40.80 ± 4.52	6.00 ± 0.75	20.40 ± 2.26
10	46.81 ± 5.20	6.88 ± 0.76	23.40 ± 2.60
30	68.21 ± 4.85	10.03 ± 0.71	34.10 ± 2.42
50	85.61 ± 3.85	12.59 ± 0.57	42.80 ± 1.92
80	113.37 ± 2.85	16.67 ± 0.42	56.68 ± 1.43
120	120.07 ± 3.69	17.66 ± 0.54	60.04 ± 1.85
180	133.35 ± 4.15	19.61 ± 0.61	66.67 ± 2.08
390	138.44 ± 6.82	20.36 ± 1.00	69.22 ± 3.41
500	119.20 ± 5.63	17.53 ± 0.83	59.60 ± 2.82

* Extractions were performed in duplicate and the results are expressed as the mean ± standard deviation (SD).
